# Mitochondrial Macular Dystrophy—A Case Report and Mini Review of Retinal Dystrophies

**DOI:** 10.3390/jcm14228236

**Published:** 2025-11-20

**Authors:** Grzegorz Rotuski, Katarzyna Paczwa, Justyna Mędrzycka, Radosław Różycki, Joanna Gołębiewska

**Affiliations:** Military Institute of Aviation Medicine in Warsaw, 01-755 Warsaw, Poland

**Keywords:** maternally inherited diabetes and deafness, retinal dystrophy, macular dystrophy, mitochondrial retinal dystrophy, multi-modal imaging, ophthalmic genetics

## Abstract

**Background**: Retinal dystrophies are often challenging to diagnose. At early stages, they may resemble benign retinal pigment epithelium alterations and drusen present in otherwise healthy individuals. With the increased incidence of autoimmunity-related disorders and new treatments for retinal dystrophies on the horizon, thorough investigations and making the correct diagnosis in time are particularly important for these patients. **Case report**: A 44-year-old myopic female was admitted to the Ophthalmology Department with a 3-week history of painless blurred vision in her right eye. Fundoscopic examination revealed the presence of optic disc edema in this eye with pigmentary and atrophic changes in the macular regions of both eyes. She had no prior ophthalmic history nor systemic comorbidities known at the time. Marked hyperglycemia and renal angiomyolipoma were discovered subsequently. Ultimately, a diagnosis of Maternally Inherited Diabetes and Deafness was made. **Discussion and Conclusion**: Maternally Inherited Diabetes and Deafness is a rare mitochondrial disorder that should be considered in the differential diagnosis of retinal dystrophies, particularly due to multi-organ syndromes they can occur with, requiring collaborative medical care of several specialists. Integrating the findings and comparing them with other online sources facilitates clinical differential and treatment selection, eventually promoting faster accurate diagnosis of patients. It is especially important because of a long waiting time for results of genetic testing, while ophthalmic pathology can be the first sign of the disease.

## 1. Introduction

Numerous diseases are linked with retinal deterioration. Based on current knowledge, these are mostly progressive defects; hence, patients presenting to ophthalmologists with vision loss often have severe damage. Autoimmunity has been identified to be implicated in this process [1,2,3,4,5], but the fact that some individuals are more predisposed invokes the need to delve deeper into the pathogenesis. Intergenic interactions are more primordial in the suspected causative chain of events and become more of a focus for current studies [6,7].

Retinitis pigmentosa (RP) is probably one of the most investigated retinal dystrophies due to its high prevalence and diversified phenotypic presentation, concerning even two million individuals worldwide at all ages [8]. The question of genetics always arises in regard to alterations linked with the environment and infectious factors, especially viruses, due to their ability to penetrate the genome, an inherent part of their reproduction when the genetic material is being translated for the host cell’s mitosis, which can cause signalling dysfunctions [9]. Therapies with correct genes mediated on unharmful viral vectors injected subretinally are currently in development, with a few specific mutations already being treatable [10]. Care needs to be taken when it comes to the pleiotropic effect of genes, since modifications can result in serious adverse effects, particularly when the defect concerns large sets, as in ABCA4 responsible for Stargardt disease among other retinal dystrophies [11]. On the contrary, in the case of age-related macular degeneration (AMD), plural genetic polymorphisms can be responsible for disease onset, and more complex therapies would be required for a change-reversing treatment [12,13]. On the other hand, mitochondrial DNA (mtDNA) mutations cause a specific group of inherited retinal dystrophies with progressive dysfunction of the retinal pigment epithelium (RPE) and photoreceptors, often accompanied by systemic manifestations due to dysfunctional mitochondria in the whole organism.

When diagnosing retinal dystrophies, photographic features are very important in making the differential diagnosis. Comparative analysis of optical coherence tomography (OCT) scans and fundus photos with databases existing in the literature is essential to select suspected genes for testing, particularly because of the cost and longitude of the process when checking for several genetic mutations [14,15]. Therefore, this study presents a case report of mitochondrial macular dystrophy with atypical presentation. Due to confounding findings throughout the diagnostic process, we reviewed the key features of inherited retinal dystrophies to demonstrate the necessary considerations, since genetic testing is costly and lengthy.

## 2. Case Report

We present the case of a 44-year-old myopic Caucasian female who was consulted at the ophthalmic emergencies in January 2025 with a three-week history of painless blurred vision in the right eye. Her medical, ophthalmic, and family history were unremarkable. Best-corrected visual acuity (BCVA) was 20/33 in the right eye and 20/20 in the left eye. Intraocular pressures (IOP) were within normal limits bilaterally. Ishihara colour vision test results were normal. A slit-lamp examination of the anterior segment revealed no abnormalities. Fundoscopic examination revealed grade 2 optic disc edema in the right eye according to the Frisen scale, as well as pigmented macular deposits accompanied by segmental atrophy in both eyes. At the time, the visual field (VF) was non-diagnostic because of a high false-negative rate. The patient was admitted to the Ophthalmology Clinic for further diagnostic evaluation.

### Investigations and Results

**Figure 1:**
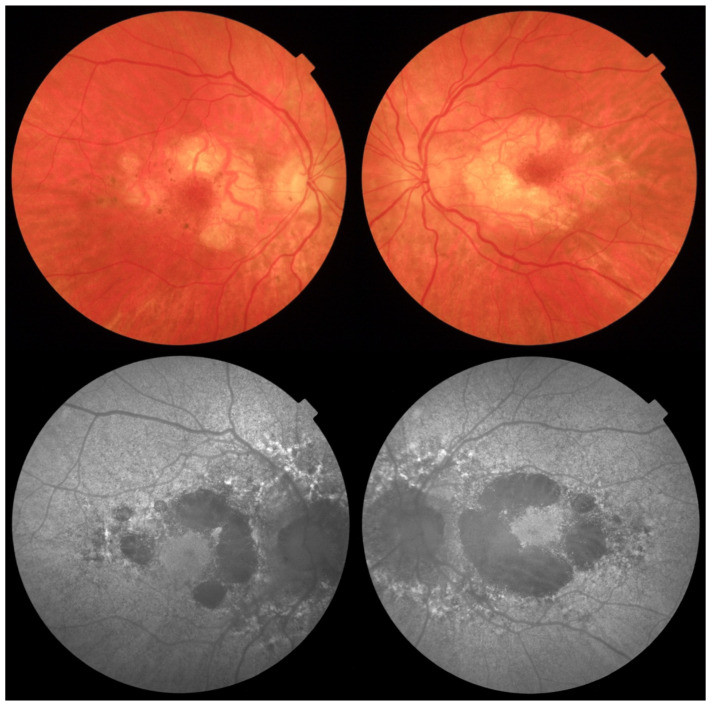
Colour fundus photography showing circular areas of RPE atrophy and small hyperpigmented deposits in the macular region of the right and left eye in horizontal and vertical B-scans, respectively. Autofluorescence shows circular areas of geographic atrophy.

**Figure 2:**
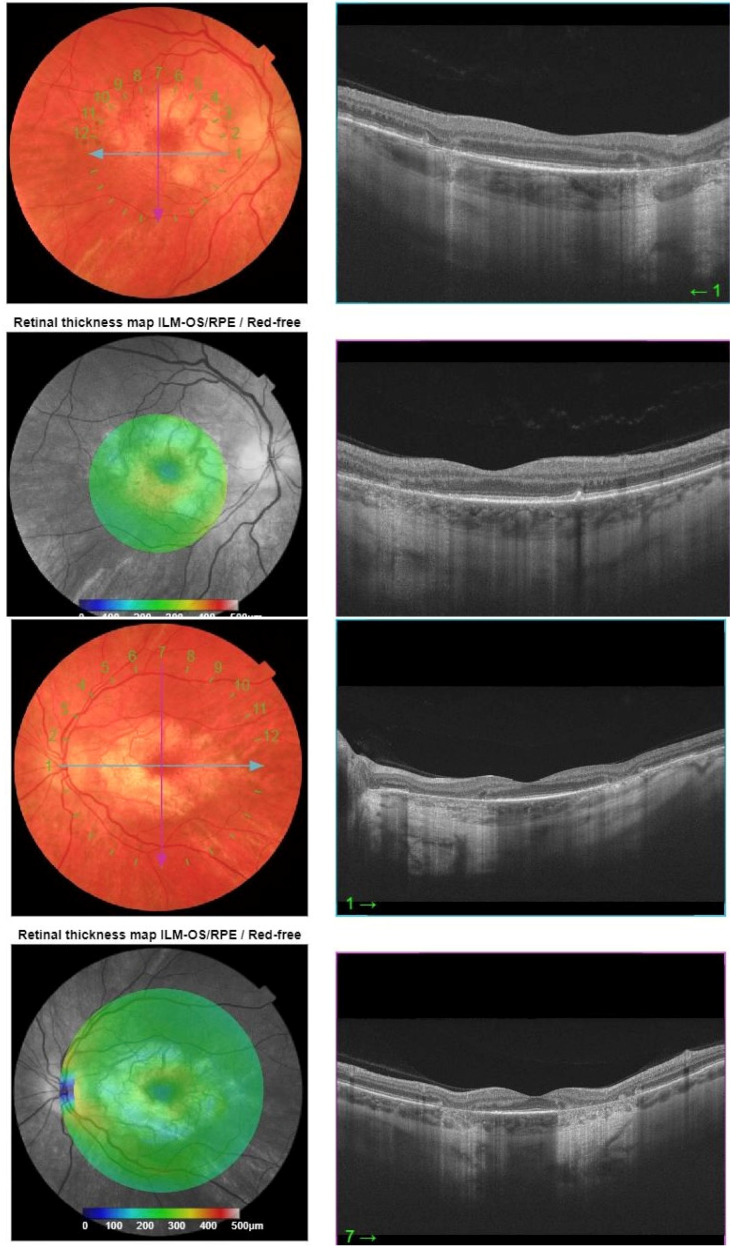
OCT B-scans with horizontal and vertical projections, respectively. The right eye shows hyperreflective deposits in the RPE layer and focal RPE atrophy, while the left eye shows focal RPE atrophy.

**Figure 3:**
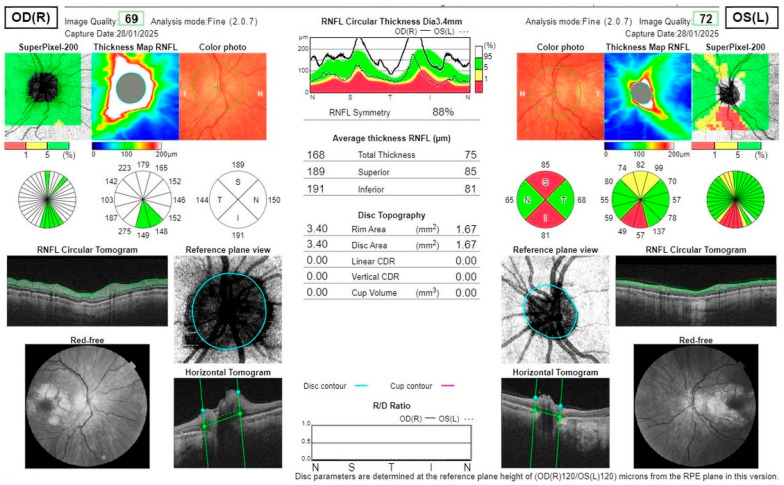
OCT scans of the RNFL show optic disc edema in the right eye and superior and inferior thinning in the left eye.

**Figure 4:**
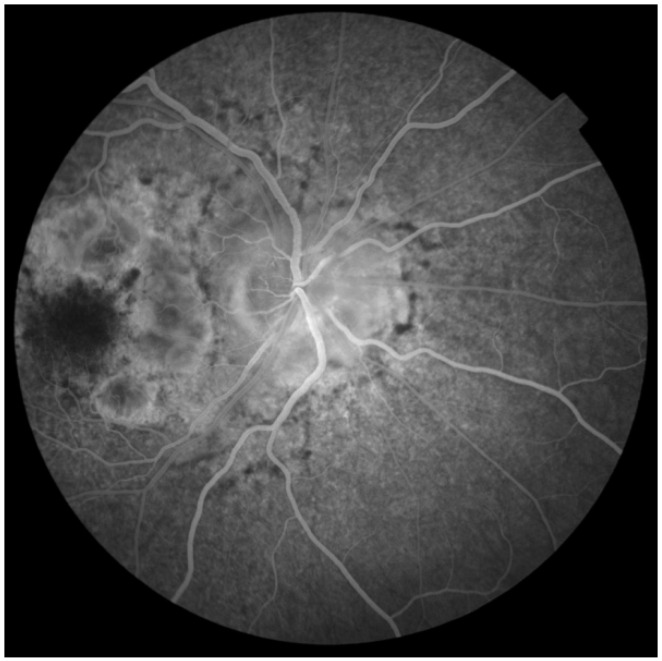
Fluorescein angiography of the right eye shows subtle leakage from the right optic disc.

Fundus autofluorescence (FAF) of the posterior pole revealed bilateral hypoautofluorescent circular areas in the macular region (Figure 1). OCT demonstrated hyperreflective deposits at the level of the RPE with focal RPE atrophy (Figure 2), optic disc edema in the right eye, and inferior retinal nerve fibre layer (RNFL) thinning in the left eye (Figure 3). Fluorescein angiography (FA) showed leakage from the right optic disc (Figure 4).

Laboratory evaluation revealed marked hyperglycemia (blood glucose 449 mg/dL) and elevated glycated hemoglobin (HbA1c) at 13.4%. Other laboratory parameters, including serologies for Borrelia, Treponema, Syphilis, and HIV, were negative. The patient was promptly initiated on insulin therapy. Maturity-onset diabetes of the young (MODY) was initially suspected. The patient’s body mass index at the time was 23.88 kg/m^2^, with a weight of 65 kg and a height of 165 cm. Family history was positive for type 2 diabetes mellitus in the patient’s grandmother. Additional tests were planned: brain and orbits magnetic resonance imaging (MRI) was normal, but abdominal ultrasound identified an angiomyolipoma of the right kidney. Creatinine and glomerular filtration levels were normal. Based on the combination of ocular manifestations, diabetes, and the renal lesion, mitochondrial diabetes and deafness (MIDD) was considered the most probable diagnosis.

Following initiation of insulin therapy in April 2025, the patient’s glycemic control improved, BCVA returned to 20/20 in both eyes, and the optic disc edema in the right eye partially resolved. Audiometry did not reveal hearing impairment. During follow-up visits, no disease progression was observed. Genetic testing for MIDD has been planned to confirm the diagnosis. The analyzed genes involved a comprehensive inherited retinal disease panel, as follows: ABCA4, ABCC6, ABHD12, ACBD5, ACO2, ADAM9, ADAMTS18, ADGRA3, ADGRV1, ADIPOR1, AFG3L2, AGBL5, AHI1, AHR, AIPL1, AIRE, ALDH3A2, ALMS1, ALPK1, AMACR, ARHGEF18, ARL13B, ARL2BP, ARL3, ARL6, ARMC9, ARMS2, ARSG, ASRGL1, ATF6, ATXN7, B9D1, B9D2, BBIP1, BBS1, BBS10, BBS12, BBS2, BBS4, BBS5, BBS7, BBS9, BEST1, C1QTNF5, C2, C2CD3, C3, CA4, CABP4, CACNA1F, CACNA2D4, CAPN5, CC2D2A, CCDC28B, CC12, CDH23, CDH3, CDHR1, CELSR2, CEP104, CEP120, CEP164, CEP19, CEP250, CEP290, CEP41, CEP78, CERKL, CFAP410, CFAP418, CFB, CFH, CHM, CIB2, CLCC1, CLEC38, CLN3, CLN5, CLN6, CLN8, CLRN1, CLUAP1, CNGA1, CNGA3, CNGB1, CNGB3, CNNM4, COL11A1, COL2A1, COL9A1, COQ2, CPLANE1, CRB1, CRX, CSPP1, CTC1, CTNNA1, CTNNB1, CTSD, CWC27, CYP4V2, DHDDS, DHX38, DMD, DRAM2, DTHD1, DYNC2H1, DYNC2I2, EFEMP1, ELOVL1, ELOVL4, EMC1, ENSA, ERCC6, ERCC8, ESPN, EXOC8, EXOSC2, EYS, FAM149B1, FAM161A, FBLN5, FBN2, FLVCR1, FRMD7, FSCN2, FZD4, GDF6, GDPD1, GNAS, GNAT1, GNAT2, GNB3, GNPTG, GPR143, GPR179, GRK1, GRM6, GRN, GUCA1A, GUCA1B, GUCY2D, HARS1, HGSNAT, HK1, HKDC1, HMCN1, HMX1, HTRA1, HYLS1, IDH3A, IDH3B, IFT140, IFT172, IFT27, IFT43, IFT74, IFT81, IKBKG, IMPDH1, IMPG1, IMPG2, INPP5E, INVS, IQCB1, ITM2B, JAG1, KATNIP, KCNJ13, KCNV2, KIAA0586, KIAA0753, KIAA1549, KIF11, KIF3B, KIF7, KIZ, KLHL7, LAMA1, LAMP2, LCA5, LRAT, LRIT3, LRP2, LRP5, LZTFL1, MAK, MAPKAPK3, MED12, MERTK, MFN2, MFRP, MFSD8, MIEF1, MIR204, MKKS, MKS1, MMACHC, MSTO1, MTRFR, MTTP, MVK, MYO7A, NBAS, NDP, NEK2, NEUROD1, NMNAT1, NPHP1, NPHP3, NPHP4, NR2E3, NR2F1, NRL, NYX, OAT, OFD1, OPA1, OPA3, OPN1LW, OPN1MW, OPN1SW, OTX2, PANK2, PAX2, PCARE, PCDH15, PCYT1A, PDE6A, PDE6B, PDE6C, PDE6D, PDE6G, PDE6H, PDSS1, PDZD7, PEX1, PEX2, PEX6, PEX7, PGK1, PHF6, PHYH, PIBF1, PISD, PITPNM3, PLA2G5, PLK4, PNPLA6, POC1B, POC5, POMGNT1, POMT1, PPT1, PRCD, PRDM13, PRKCD, PROM1, PROS1, PRPF3, PRPF31, PRPF4, PRPF6, PRPF8, PRPH2, PRPS1, RAB28, RAX2, RB1, RBP3, RBP4, RCBTB1, RD3, RDH11, RDH12, RDH5, REEP6, RGR, RGS9, RGS9BP, RHO, RIMS1, RIMS2, RLBP1, RNU4ATAC, ROM1, RP1, RP1L1, RP2, RP9, RPE65, RPGR, RPGRIP1, RPGRIP1L, RRM2B, RS1, RTN4IP1, SAG, SAMD11, SCAPER, SDCCAG8, SEMA4A, SGSH, SIX6, SLC24A1, SLC25A46, SLC4A7, SLC6A6, SLC7A14, SNRNP200, SPATA3, SPATA7, SPP2, SRD5A3, STN1, SUFU, TCTN1, TCTN2, TCTN3, TEAD1, TIMM8A, TIMP3, TINF2, TLCD3B, TLR3, TLR4, TMEM107, TMEM126A, TMEM138, TMEM216, TMEM218, TMEM231, TMEM237, TMEM67, TOPORS, TPP1, TRAF3IP1, TREX1, TRIM32, TRNT1, TRPM1, TSPAN12, TTC21B, TTC8, TTLL5, TIPA, TUB, TUBB4B, TUBGCP4, TUBGCP6, TULP1, UNC119, USH1C, USH1G, USH2A, USP45, VCAN, VPS13B, WDPCP, WDR19, WFS1, WHRN, YPEL2, ZFYVE26, ZNF408, ZNF423, ZNF513.

The NGS-based panel analysis identified carriage of the pathogenic m.3243A > G variant in the MT-TL1 gene (with 14% heteroplasmy percentage, measured in the blood sample). This variant is associated with mitochondrial disorders, including Mitochondrial Encephalopathy, Lactic Acidosis, and Stroke-like Episodes (MELAS), as well as Maternally Inherited Diabetes and Deafness (MIDD). Moreover, a variant of unknown significance (VUS) was detected in c.5124-3C > G of the CACNA1F gene at 40% according to the Association for Clinical Genomic Studies (ACGS) guidelines. The patient subsequently underwent a clinical genetics consultation, during which additional testing was recommended, including her family members. The geneticist instructed to analyze the heteroplasmy for MT-TL1 in other tissues when performing a muscle biopsy, which would help the differential diagnosis. At this point, she is reluctant to undergo further genetic testing and stays under the close supervision of our Ophthalmology Team and her primary care physician.

## 3. Discussion

MIDD is a rare mitochondrial disorder that includes a characteristic pattern of retinal dystrophy in ophthalmic examination–circular atrophy surrounding the fovea and optic disc [16]. It spreads bilaterally with central involvement in advanced stages, explaining the reason for the relatively late presentation of patients to ophthalmologists. Patients can experience metamorphopsia, nyctalopia, photophobia, colour vision changes, or other nonspecific visual symptoms due to disturbance of visual information processing and disintegration of retinal layers [17]. MIDD retinal dystrophy may resemble inherited macular dystrophies and is often underdiagnosed. Vessel attenuation, more commonly associated with retinitis pigmentosa, can also be a feature in the late stages due to atrophic changes [18]. OCT may reveal outer retinal thinning and disruptions of the ellipsoid zone. Performing fundus autofluorescence shows a mottled appearance of the retina and is a highly suggestive, fast, and easy examination to narrow down possible causes of vision loss. Optic disc edema is not a typical finding, though it has been described in some cases of retinal dystrophies [19]. In our patient, it can be argued that optic disc edema is due to uncontrolled DM. However, diabetic papillopathy would typically coexist with other symptoms such as macular edema, retinal hemorrhages, or ischemic zones evidenced on FA, which were not observed in this case. The patient was likely adapted to hyperglycemia since she presented no general symptoms, including polydipsia, polyuria, dry mouth, tiredness, weight loss, etc. The left RNFL seems to have already been damaged, and probably exacerbation of the disease in the right eye, correlated with the papilledema, occurred when the patient finally noticed visual deterioration. Optic disc edema is also found in retinal dystrophies like RP [20] and other inherited mitochondrial disorders at disease onset, such as Leber hereditary optic neuropathy (LHON) [21].

Other than early-onset diabetes mellitus (2nd–4th decade, usually non-insulin-dependent), systemic features of MIDD comprise sensorineural hearing loss (cochlear hair cells damage) and possible involvement of many other organs: muscular weakness and exercise intolerance; cardiomyopathy with arrhythmia or heart block; gastrointestinal dysmotility; renal glomerulosclerosis; and, most debilitating, central nervous system disorders, including seizures, migraines, stroke-like episodes, basal ganglia calcification, cerebellar atrophy, encephalopathy, and developmental delay [22,23,24]. Renal angiomyolipoma is not a typical finding, but along with DM and macular dystrophy, it can be clinically framed with the suspected mitochondrial disease, since the metabolism of the kidneys, pancreas, and retina depends on ciliated cells [25]. The analysis of TSC1 and TSC2 genes in this case could help in further differential diagnosis, as they are associated with the tuberous sclerosis complex disorder. They code for proteins hamartin and tuberin, involved in controlling cell growth and division; therefore, if impaired, the risk of cancerogenesis increases [26]. Abnormalities involve skin lesions and pigmentary changes, brain malformations, heart rhabdomyomas, and lung lymphangioleiomyomatosis [27]. At this point, no other systemic finding nor typical phenotype was identified in the context of tuberous sclerosis in this patient.

Additionally, in MIDD and other mitochondrial disorders, there is a higher risk of acute hepatic failure due to reduced tolerance of drug metabolism resulting from impaired hepatocytes [28]. Hair follicles and dermal fibroblasts are also weakened; hence balding or pigmentary skin changes can be observed [29]. Mitochondrial disorders are essentially multi-systemic diseases, where progressive failure of particular organs occurs at different time points. In children, these disorders are more debilitating since the damage accumulates over the years and their bodies may not mature correctly, making them more prone to reduced energy for growth and remodelling of bones, leading to short stature and early osteoporosis, which can be further exacerbated by impaired growth hormone production and thyroid dysfunction [30]. This shows the importance of multidisciplinary coordinated medical care for these patients, although the full phenotype may not be present due to variable expression of genes among individuals. Mitochondrial dysfunction leads to defective ATP production and the inability to counteract oxidative stress, with accumulation of reactive oxygen species and other toxic metabolic waste that damages cells, and then progressively tissues, resulting in impaired photoreceptors and RPE survival [31,32]. It can derive from mtDNA point mutations, deletions, or duplications, but also alterations in nuclear-encoded mitochondrial genes (e.g., POLG, TWNK, HSPA9) [33]. MIDD itself is most often caused by the mutation A3243G within the MT-TL1 tRNA (Leu (UUR)), but many other possible genes in mtDNA can be responsible for this disease [34]; therefore, an experienced geneticist should help choose adequate investigations. The heteroplasmy in the patient’s pathogenic m.3243A > G variant at 14% is relatively low for the certainty of making the adequate clinical diagnosis, with levels exceeding 40–90% being more coherent [35]. Muscle biopsy or urine sedimentation tissue analysis could enhance the knowledge of the patient’s prognosis upon detection of a higher heteroplasmy percentage.

Apart from mitochondrial disorders, these patients present with deficits in high-energy-demanding organs; clinical suspicion is based on family history–typically maternal inheritance–which may be negative since mutations in DNA can happen spontaneously [36]. Genetic testing for the mutation can be performed from a blood sample or buccal swab, but the gold standard is muscle biopsy for high heteroplasmy detection [37]. Despite advances in gene therapy and mitochondrial editing, current management is mostly supportive with avoidance of mitochondrial toxins (aminoglycosides, valproic acid, linezolid, alcohol, smoking) [38,39] and supplementation of idebenone and antioxidants (CoQ10, vitamin C&E, resveratrol, NAD+, etc.) [40,41,42,43]. Idebenone is a short-chain benzoquinone (C19H30O5) registered for the treatment of Leber Hereditary Optic Neuropathy (LHON), which acts by directly donating electrons to complex III of the mitochondrial respiratory chain, bypassing the dysfunctional complex I, enabling restoration of ATP production through oxidative phosphorylation [44]. However, not all LHON mutations react favourably to this treatment, so caution and careful monitoring are advised and more research is necessary. Experimental therapies include mitochondrial replacement techniques to avoid transmission to offspring [45], allotopic gene therapy [46], and stem cell approaches, including induced pluripotent stem cells [47]. Visual rehabilitation is provided in cases with severe visual deterioration–same goes for hearing aids if necessary. Metformin, the drug of choice in diabetic patients, should be avoided to combat hyperglycemia due to the increased risk of lactic acidosis in mitochondrial diseases [48].

As for the CACNA1F gene mutation, it is related to X-linked retinal disorders, such as congenital stationary night blindness, cone-rod dystrophy, and Åland Island eye disease, all connected with a calcium channel dysfunction in retinal photoreceptor synapses [49]. In our patient’s genetic testing, it was marked as a VUS, meaning its pathogenicity is unknown.

The yet uncompleted genetic testing in the patient’s family members is a limitation of this study. With incoherent findings in the patient herself, analyzing the genetic and phenotypic presentation of the maternal relatives in particular could provide more reassurance on the correct diagnosis. Still, more research is warranted to understand the gene-phenotype correlations for specific genes and their pathogenicity depending on factors of resurgence, when other individuals with the same mutation remain asymptomatic.

### Classification of Retinal Dystrophies

In order to reach a definite diagnosis of MIDD, understanding the complexity of inherited retinal disorders is necessary. They should be divided into general subgroups, then associated with clinical findings from the most prevalent to the rarest. Photoreceptor dystrophies are among the most common types of retinal genetic disorders. They can have higher predilection to rods (RP, congenital stationary night blindness) or to cones (achromatopsia, blue cone monochromacy) [50,51]. They may affect primarily the macular area (Stargardt, Best, North Carolina, Sorsby, Doyne honeycomb dystrophy) or the entire retina (choroideremia, gyrate atrophy, Bietti crystalline dystrophy, Goldmann-Favre Syndrome) [52]. Some dystrophies are vitreoretinal disorders (X-linked Retinoschisis, Familial Exudative Vitreoretinopathy, Stickler Syndrome, Wagner Syndrome) [53], others can selectively affect a particular retinal layer, e.g., pattern dystrophies (Adult-onset Foveomacular Vitelliform Dystrophy, Butterfly-shaped Pigment Dystrophy, Reticular RPE Dystrophy or Oguchi Disease) [54]. A subgroup resulting from mitochondrial dysfunction, comprising MIDD, is distinguished: LHON, Mitochondrial Encephalopathy with Lactic Acidosis and Stroke-like Episodes (MELAS), myoclonic epilepsy with ragged red fibres (MERRF), NARP Syndrome (Neuropathy, Ataxia, and Retinitis Pigmentosa), Chronic Progressive External Ophthalmoplegia (CPEO), and Kearns-Sayre Syndrome [55].

Initially, in suspicion of inherited retinal disease, a work-up of the most characteristic genetic mutations should be performed, in relation to the population where they are observed. A useful first step is making a family tree diagram, with inspection of their known ophthalmic issues, ethnical background of close members, and potential consanguinity of direct ascendants that dramatically increases the risk of genetic disorders in the offspring. Based on the appearance of the retina or examinations already performed by the patient prior to the consultation, the most prevalent genes in the patient’s population should be considered to plan the diagnostic process carefully, in view of the longitude of genetic testing and the cost-effectiveness. Binocular peripheral retinal changes should bring in mind RP, which can present with various phenotypes considering many possible gene mutations (RPE65, RPGR, USH2A, MERTK, PDE6B). In the case of maculopathies, primary considerations should concern genetic mutations in PRPH2 (=RDS), BEST, PROM1, CRX, and dominant macular dystrophy. Since inherited retinal dystrophies are a group of monogenic disorders, they can be classed into syndromic and non-syndromic.

Syndromic:

- PYGM—muscle glycogene phosphorylase, so they are tired soon after exercise
- DMPK—myotonic dystrophy
- ABCC6—angioid streaks (connective tissue disorders, hemoglobinopathies)
- CLN7/MFSD8—neuronal ceroid lipofuscinosis = Batten disease
- MIDD

And non-syndromic (typically without systemic manifestations):

- PRPH2—can induce various retinopathies
- BEST1—bestrophinopathies
- IMPG1/2—vitelliform macular dystrophy
- ABCA4—Stargardt disease
- CTNNA1—butterfly-shaped pigment dystrophy.

What should be included in the differential diagnosis of MIDD, particularly at early stages, involves Stargardt disease, AMD, RP characterized by diversified phenotypes and pattern dystrophies, along with drug-induced retinopathies. Rhodopsin, the pigment present in rods, gives off toxic metabolites in RP, but cones are affected in late stages, so visual changes are noticed in advanced disease, much like in typical glaucoma with progressive peripheral vision loss. On FAF, nummular atrophy of the RPE in the peripheral retina can sometimes be observed, correlated with particular gene mutations. Other RP findings include intraretinal macular cysts on OCT B-scans and posterior subcapsular cataract formation. Due to the multifactorial genetic background of RP, ophthalmologists should be familiar with the differences between autosomal dominant, autosomal recessive, and X-linked phenotypes, to provide thorough counselling regarding the prognosis for the patients, their relatives, and perspectives of transmission to offspring. In Stargardt disease, however, cones are affected first, so children and adolescents are referred to ophthalmologists early, with characteristic eye fundus midperipheral pisciform flecks spreading peripherally. Another suggestive finding is visual improvement after restricting the diet from vitamin A products due to their impaired metabolism. ABCA4 gene mutations, comprising Stargardt disease, present with peripapillary sparing, which is useful information to discern MIDD. Due to retinal dystrophies often initially presenting with bull’s eye maculopathy, it is important to understand this entity [56]. It represents retinal dysfunction at early stages of involvement but is non-specific and needs to be correlated with medical history. Upon discovery, toxic and metabolic diseases need to be excluded firsthand. Drugs responsible for this adversity include chloroquine analogues (typically used in the treatment of autoimmune disorders–the risk increases exponentially with higher doses and duration of intake), desferrioxamine (anemia), pentosan polysulfate sodium (interstitial cystitis), and thioridazine (once used for schizophrenia, now uncommon in clinical practice). With the advent of new biologic therapies being constantly developed, particularly in oncology and neurology, awareness should be taken to review any reported drug toxicity caused by these innovative drugs. On the other hand, metabolic disorders concern lysosomal storage diseases (MPS, Tay-Sachs, gangliosidosis, Batten disease), leading to lipofuscin overload, meaning cellular metabolic waste aggregates in the outer retina, harming photoreceptors. After excluding these relevant causes of bull’s eye maculopathy, local retinal sources of toxicity should be inspected.

**Figure 5:**
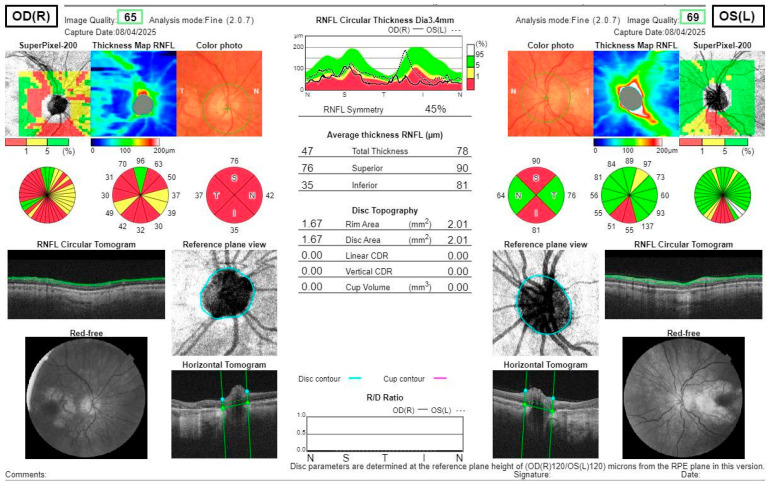
Post-treatment OCT scans show the RNFL thickness reduction in all quadrants in the right eye, with no optic disc edema and superior and inferior thinning in the left eye.

Pattern dystrophies become much more apparent upon performing FAF, which consists of exciting the lipofuscin localized under the retinal bed and obtaining lower reflectivity (dark spots) in areas of RPE defects, causing characteristic patterns [57]. OCT B-scans are also very helpful to demonstrate the particular layers affected, as shown in our case (Figure 3, Figure 4 and Figure 5) with outer retina loss. Electroretinography (ERG) is a useful tool in differentiating retinopathies from neuropathies, the latter more typically causing an impaired response in visually evoked potentials (VEP) [58]. Electrophysiological findings can be misleading since, for instance, full-field ERG is often normal unless extensive involvement of the retina is present, though lowered responses with disproportionate structural damage may orient the diagnosis to acute causes such as intoxication [59]. One has to be mindful of systemic associations related to retinopathies, starting with examination of ocular adnexa and motility (ptosis, myopathy, and cardiomyopathy in mitochondrial disorders) [60]. Family history should be taken into account and lab tests performed (blood/urine lactate, muscle biopsy, CSF analysis), with MRI performed for CNS involvement [61]. Often, primary presentation concerns solely ophthalmic findings due to the high energy demand of retinal tissue–especially the photoreceptor/RPE complex, but also upper eyelids position due to the constant need to keep the eyes open through daily activities by Muller’s muscle contraction [62].

Multi-modal imaging, meaning the use of several modalities to grasp the discerning features in the targeted tissues, is very relevant to narrow down possible diagnoses by comparing images obtained through different technologies. For instance, in the case of our patient, we combined OCT, FAF, FA, VF, MRI, and ultrasound to discover both characteristic and atypical findings for MIDD. Artificial intelligence in retinal image analysis could help in the differential diagnosis by swiftly comparing the clinical cases available in online databases, with a selection of similar pictures to check out the confirmed diagnosis [63,64,65]. However, in terms of inherited retinal diseases, there are several issues with this seemingly straightforward strategy, such as a degree of phenotypic variability that can give an erroneous association based on retinographic findings [66,67,68]. On top of that due to pleiotropism of genes, two parallel retinopathies may be overlapping, blurring the diagnostic picture [69]. As for mtDNA, it is tough to extract and analyze [70]. An important piece of awareness should be brought to the fact that Sanger whole genome-sequencing (WGS) may not be enough, since mutations can happen in the other allele of the gene subjected to examination [71]. Sometimes, exome panels are a useful addition [72,73]. Long-read sequencing and heteroplasmy (presence of plural mtDNA variants) mapping with quantification taking threshold effects (mutation percentage causing a disease) into account should be considered [74,75]. Array genomic hybridization (AGH) can identify chromosomal gains or losses much smaller than those depicted by commonly performed karyotyping or chromosome banding [76], which could shed light on the interpretation of variants of unknown significance (VUS).

After overcoming the diagnostic burden, there remains the deception of having no solid treatment solution for most retinal dystrophies. Due to the limited regeneration of retinal tissues, replacement strategies are one of the possibilities and have been tested for years. Cultivation of correctly layered, interconnected retinal layers has been achieved [77,78], but incorporating the obtained organoid structure into the visual pathway still gives poor results, similar to various retinal electronic implants tested in parallel [79]. Challenges to overcome include stimulating brain neuroplasticity to recover damaged transmission pathways with connections to all brain lobes for full visual processing, while avoiding oncogenicity risk at the same time. In primary prevention or stopping progression early, genetic therapies will be crucial, in order not to result in repeat retinal deterioration after a potential tissue transplantation, but thorough testing is significant to avoid serious adverse effects, being more probable when targeting the genome [80]. Ultimately, in order to prevent the senescence of cells, telomerase may be targeted as an option–research shows it could slow down brain ageing [81] and thus also prevent ocular degenerative diseases, due to the eyes being essentially a part of the central nervous system.

## 4. Conclusions

Case reports remain valuable in expanding the understanding of phenotypic variability among retinal diseases, while also systematizing knowledge around the topic of interest. Recognizing and understanding the photographic features of retinal diseases using multimodal imaging is very useful and important, but further correlation with functional tests and lab work-up is often necessary. Future perspectives show the rising importance of genetics in ophthalmology–retinology in particular, and the need for closer collaboration between these specialties, with mutual understanding of the basics in both fields for new breakthroughs. Investing in international medical registries for rare diseases can help facilitate managing patients and finding new solutions to help them.

Furthermore, this case highlights the crucial necessity of avoiding metformin in patients with suspected or confirmed mitochondrial diabetes (MIDD) due to the significantly increased risk of lethal lactic acidosis.

## Data Availability

Data are available from the corresponding author only at the justified request of a scientist.
